# Whole genome sequencing of CRISPR/Cas9-engineered NF-κB reporter mice for validation and variant discovery

**DOI:** 10.1038/s41597-024-04064-8

**Published:** 2024-11-13

**Authors:** Guruswamy Mahesh, Erik W. Martin, Mohammad Aqdas, Kyu-Seon Oh, Myong-Hee Sung

**Affiliations:** 1 Transcription Systems Dynamics and Biology Unit, Laboratory of Molecular Biology and Immunology, National Institute on Aging, National Institutes of Health, 251 Bayview Boulevard, Baltimore, MD 21224 USA; 2 Present Address: Booz Allen Hamilton, 8283 Greensboro Drive, McLean, VA 22102 USA

**Keywords:** Inflammation, Cellular imaging

## Abstract

Targeted knockout, mutations, or knock-in of genomic DNA fragments in model organisms have been used widely for functional and cell-tracking studies. The desired genetic perturbation is often accomplished by recombination-based or CRISPR/Cas9-based genome engineering. For validating the intended genetic modification, a local region surrounding the targeted locus is typically examined based on enzymatic cleavage and consequent length patterns, e.g. in a Southern analysis. Despite its wide use, this approach is open to incomplete and ambiguous readouts. With decreasing costs of high-throughput sequencing, it is becoming feasible to consider a large-scale validation of a new strain after a targeted genetic perturbation. Here we describe a dataset of whole-genome sequences and the variant analysis results from four novel reporter mouse strains. This served to validate the strains and identified all the off-target effects on the genome, thereby increasing the genetic diversity of genomic sequences over those represented in the public databases for inbred mice.

## Background & Summary

Genomic engineering has become a widely used tool for conducting biological research. It is also increasingly being used in the clinic to deliver groundbreaking treatments to patients in need. A particularly powerful approach to genomic engineering is the incorporation of exogenous DNA sequences into a host’s native genome via targeted integration^1^, whereby the integrated DNA can be regulated by endogenous promoters and other native elements. Targeted integration can serve both medical and research purposes including inserting engineered T-cell receptors (TCRs) into native TCR loci of primary T cells to create TCR-T immunotherapies to combat cancer (a.k.a. orthotopic TCR replacement)^2^; correcting defective genes such as HBB (hemoglobin subunit beta) to treat sickle cell disease^3^; and adding traceable or fluorescent tags to genes and subsequently the proteins they produce to interrogate and quantify the biological functions of the tagged proteins^4^.

The targeted integration at site-specific locations can be achieved via sequence homology-based DNA repair mechanisms (homologous recombination)^5^ after cleavage of the host chromatin at the exact site where the exogenous DNA is to be inserted into the genome. The template for homologous recombination and exogenous DNA insertion is typically linear single-stranded DNA or double-stranded DNA (linear or circular), often with the exogenous DNA sequence flanked by the host DNA sequences surrounding the specific cleavage and DNA insertion site. The template can also be produced via reverse transcription of RNA as in the prime editing approach^6^. The cleavage of site-specific DNA sequences at which the exogenous DNA is to be integrated is typically carried out through use of nucleases such as transcription activator-like effector nucleases (TALENs)^7^; zinc-finger nucleases (ZFNs)^8^; and clustered regularly interspaced short palindromic repeats (CRISPR)-Cas-associated nucleases^9^. Among these, the recent CRISPR/Cas-based approaches have become the most preferred means of targeted integration.

Verifying successful targeted integration of exogenous DNA into the host genome is a critical step in validating the suitability of a drug product or resource produced for research purposes. In the case of research-grade resources, such validation is often achieved by investigating the local region surrounding and including the targeted locus: enzymatic cleavage and consequent length patterns of the non-engineered and engineered loci are compared, e.g., in a Southern analysis. Yet, despite its widespread use, this approach is relatively qualitative and open to incomplete and ambiguous readouts of the modified genomic region. Moreover, it is unable to characterize off-target perturbations distant from the intended DNA insertion site. With decreasing costs of high-throughput sequencing, it is becoming cost-effective to consider large-scale, whole genome sequencing (WGS)-based validation of a new cell line or model organism strain after a targeted genetic perturbation. It is also increasingly becoming feasible to process and analyze WGS data with relatively straightforward bioinformatics tools or support. In contrast to Southern analysis, WGS provides extensive base-by-base sequence readouts of the targeted area essentially indicating exactly where the integration of exogenous DNA occurred and whether it occurred with fidelity. WGS analysis can also detect off-target integrations of the exogenous DNA and genomic perturbations resulting from off-target cleavage reactions (e.g., single-nucleotide variations (SNVs), insertions/deletions (INDELs), and translocations) elsewhere in the genome. The validation of drug products for clinical trials and FDA regulatory processes may involve variations of such comprehensive validation analyses. Yet, no single standard approach seems to be performed or required as the field is still in its relative infancy and a one-size-fits-all approach may not be appropriate. Similarly, besides Southern analysis, no standard or widely adhered to practice exists for validating targeted insertions in research settings.

**Fig. 1:**
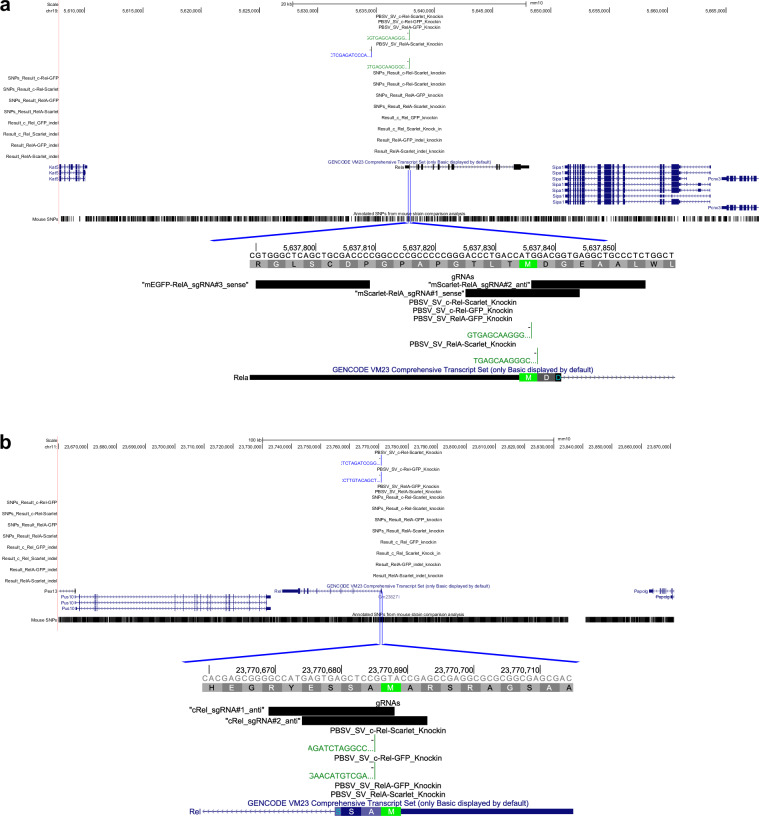
Whole-genome sequencing confirms the correct integration of targeted knock-in reporters. (**a**) A genome browsershot of the Rela locus shows the insertion of mEGFP and mScarlet sequences at the desired location at the start of the gene for the mEGFP-RelA and mScarlet-RelA knockins (but not for the other knock-ins), respectively. The lower track under the blue lines shows the zoomed-in view that includes the location of gRNAs. (**b**) A genome browsershot of the Rel locus shows the insertion of mEGFP and mScarlet sequences at the desired location at the start of the gene for the mEGFP-cRel and mScarlet-cRel knockins (but not for the other knock-ins), respectively. The lower track under the blue lines shows the zoomed-in view that includes the location of gRNAs.

Recently, for research purposes, we created four novel fluorescent reporter mouse strains utilizing CRISPR/Cas9-facilitated homologous recombination^10^ to better understand the function of the transcription factor NF-κB, a core regulatory hub underpinning various activities of the immune system. To do so, we performed targeted integration of DNA sequences encoding either of the fluorescent proteins, mEGFP or mScarlet, into the start codon of the RelA gene or the Rel gene to essentially create mice expressing mEGFP- or mScarlet-NF-κB fusion proteins from the endogenous loci encoding RelA or c-Rel (Fig. 1). These reporter mice are invaluable resources, as the mice express native levels of the NF-κB fusion proteins under the native regulatory chromatin environments. Unlike other available tools, they enable the visualization and quantification of abundance and tracking the real-time dynamics of endogenous RelA and c-Rel in live primary cells and in intact tissues, as they translocate in and out of the nucleus in response to various cellular stimuli^10^. We had initially used Southern analysis to check the integrations in the novel mouse strains.

**Fig. 2:**
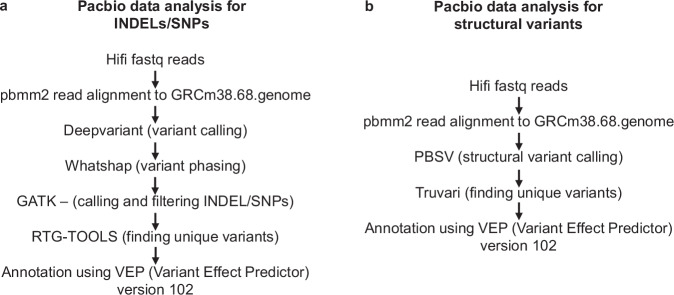
Identification of variants unique to the new strains. PacBio sequencing data analysis for INDELs/SNPs (**a**) and for structural variants (**b**). VEP (Variant Effect Predictor) predicts the effect of SNPs, INDELs, SNVs or structural variants on genes, transcripts, and protein sequence, as well as regulatory regions.

As the mice are now available to researchers worldwide and being utilized for numerous investigations involving NF-κB including its roles in immunity, disease, and aging^10,11^, we sought to validate the mouse strains more thoroughly. We performed a comprehensive analysis of the genomic-engineered mouse strains by WGS of each strain. The WGS dataset allowed variant calling analysis, when aligned to publicly available reference genomic sequences (Fig. 2). Four independent reference sequences for the wildtype C57BL/6 and two reference sequences for the other wildtype DBA/2J were compiled to ensure the novelty of variants, i.e. sequences that were detected in a reporter strain but absent in all publicly available wildtype WGS samples. We present the variant calling analysis workflow (Fig. 2) and the results, which may serve as a reference and general framework for researchers to validate genetic modifications in their own engineered cell lines, primary cells, and model organisms.

**Fig. 3:**
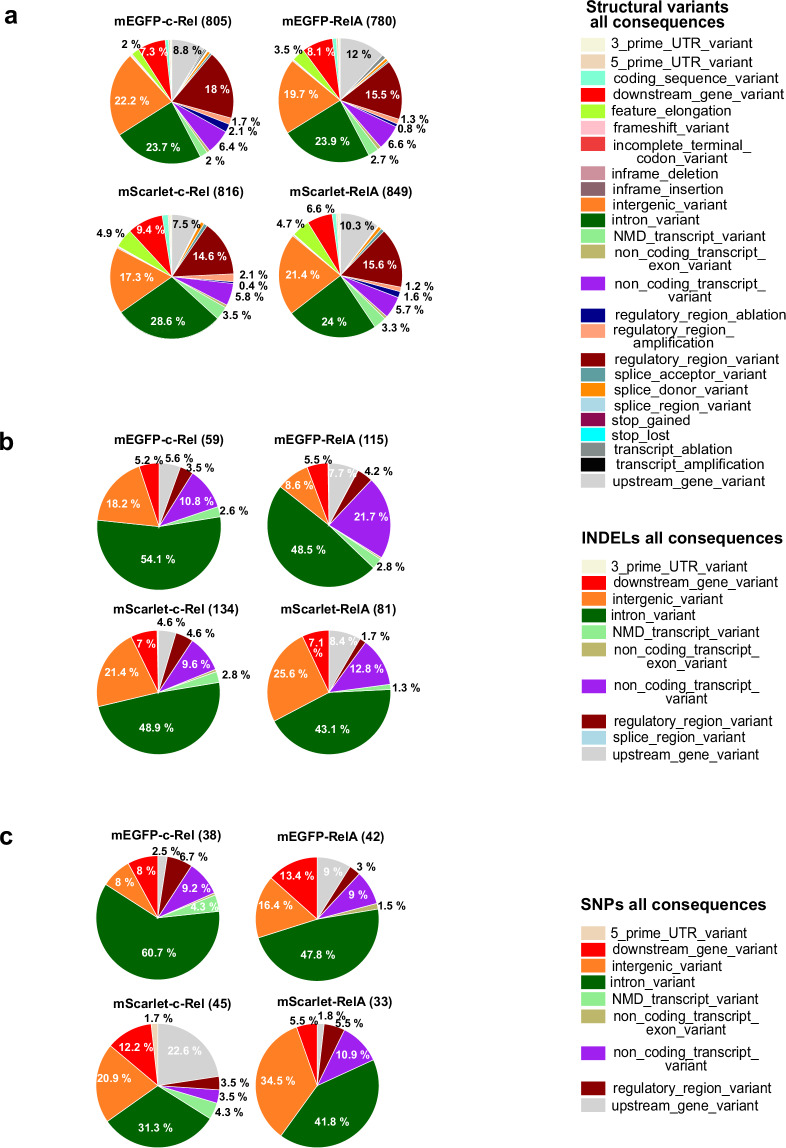
Occurrence and genomic distribution of the knock-in strain-specific variants. Pie charts show categories of structural variants (**a**), insertions/deletions (**b**), and SNPs (**c**), based on the location and type for each of the four KI strains, mEGFP-RelA, mScarlet-RelA, mEGFP-cRel, and mScarlet-cRel. Numbers in parentheses for each strain indicate the number of variants found. NMD is Nonsense Mediated Decay. Structural variants are insertions, deletions, inversions, or duplications that are larger than 50 bps.

**Fig. 4:**
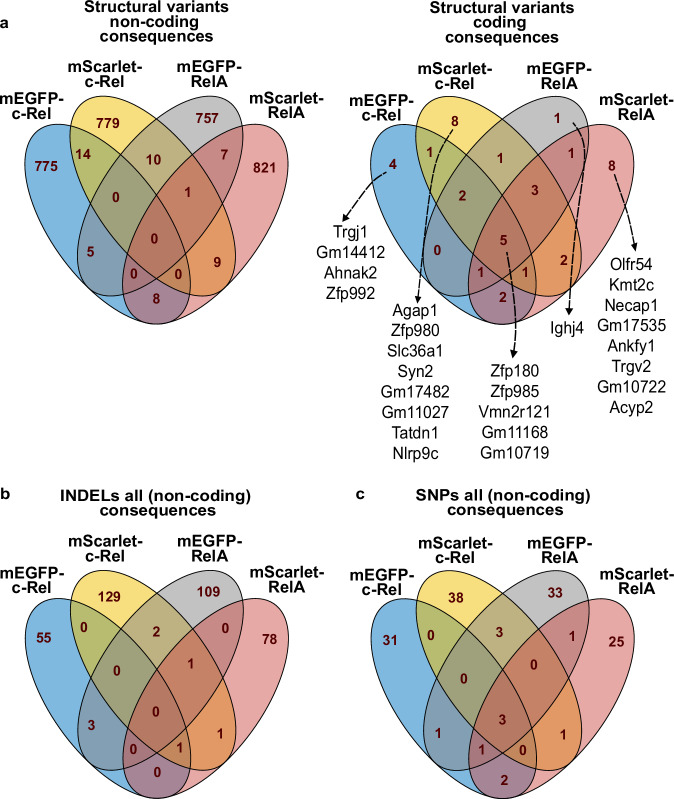
Comparison of the structural variants, INDELs, SNPs found in the four KI reporter strains. The Venn diagram shows the shared variants as well as unique variants between the four KI mouse strains, for structural variants, non-coding and coding (**a**), INDELs (**b**), and SNPs (**c**). In (**a**), gene names are shown for unique coding structural variants observed for each strain. For INDELs and SNPs, all the variants were non-coding, hence no Venn diagrams are shown for coding variants.

This analysis enabled a complete characterization of the off-target impacts of the genome engineering process. The genome sequences of the novel fluorescent reporter strains confirmed that there were few unintended genetic disruptions that were predicted to be detrimental (based on variant effect predictor (VEP) analysis; see Methods), when compared to published control sequences from wildtype C57BL/6 and DBA/2J (Figs. 3,4, Supplementary Table S1–S4). Notably, there were no overlaps between the observed variants and the candidate off-target sites predicted by Cas-OFFinder (up to 5 mismatches and 1 DNA/RNA bulge). Given the interconnected network of NF-κB and the negative and positive feedback genes, it was also reassuring that no genetic modifications were found in Nfkbia, Nfkbib, Nfkbie, Tnfaip3, Relb, Nfkb1, and Nfkb2 (Figure S1, VCF files). In particular, Nfkb1 was preserved in all four reporter strains, even though it harbors four predicted off-targets. These results are consistent with the phenotype of these mice which is indistinguishable from the wildtype.

Lastly, our WGS data may possibly contain subtle non-coding variants that may be of interest to some groups, while we verified that the novel mouse lines do not carry severe genetic disruptions. Some non-coding variants may have serendipitously perturbed genetic elements or epigenetic sites of interest to some researchers. Some of the modifications may influence DNA methylation, transcription factor binding, enhancer architecture, or genome-folding, in a tissue-specific manner.

## Methods

### Generation of knock-in mice

The four knock-in (KI) reporter mice (mEGFP-RelA, mEGFP-cRel, mScarlet-RelA, mScarlet-cRel) were generated by integrating the coding sequence of mEGFP or mScarlet into the start codon of the endogenous Rela or Rel loci using CRISPR/Cas9-based DNA editing and repair, as previously described^10^. Briefly, sgRNAs proximal to the start codon of Rela or Rel were designed using the MIT CRISPR Design online tool (crispr.mit.edu by the Feng Zhang group at MIT in 2016^12^, no longer available. Similar online tools are currently available such as the Broad Institute GPP sgRNA Designer CRISPick or E-CRISP.org). Double stranded DNA (dsDNA) donor template vectors encoded the fluorescent protein coding sequence and flanked by left and right homology arms complimentary to the native DNA sequences (including 3′ UTR and introns) surrounding the start codons of Rela or Rel (purchased from Genewiz) (.dna files available upon request). DNA sequences for HincII (Rela, mScarlet-cRel) or EcoRV (mEGFP-cRel) (for Southern analysis) and loxP sites (for Cre-based removal of the fluorescent proteins and subsequent knock-out of Rela or Rel) were added to the flanking DNA sequences. Cas9 mRNA, the respective sgRNA and dsDNA template were microinjected into one-cell stage zygotes generated from a cross between C57BL/6Ncr and B6D2F1/J mice. Founder mice containing correct targeted integrations and Rela or cRel fusion proteins were screened through collection and culturing of tail-clip fibroblasts followed by live-cell fluorescent microscopy. Functional integration and expression of fusion proteins were detected via the nuclear translocation of mEGFP- or mScarlet-RelA or -cRel fusion proteins following TNF-α stimulation. Mice determined to contain the desired integrations were then genotyped and backcrossed with C57BL/6J (IMSR_JAX:000664) mice for at least two generations.

### sgRNAs

mEGFP-RelA: “EGFP-Rela sgRNA#3” (internal tracking ID) CGTGGGCTCAGCTGCGACCC

mScarlet-RelA: “mScarlet-Rela sgRNA#1 (sense)” GACCCTGACCATGGACGGTG (PAM = AGG)

“mScarlet-Rela sgRNA#2 (antisense)” GAGGGCAGCCTCACCGTCCA (PAM = TGG)

mEGFP-cRel and mScarlet-cRel:

“c-Rel sgRNA#1 (antisense)” CCGGTACTCACTCGAGGCCA (PAM = TGG)

“c-Rel sgRNA#2 (antisense)” ACTCACTCGAGGCCATGGCT (PAM = CGG)

### Sequencing

Spleens were harvested from 10–42 week-old male KI reporter mice after each strain was backcrossed to C57BL/6J and bred to reach homozygosity for the targeted knock-in reporter. DNA was isolated for each KI strain. PacBio SMRT platform was used for long-read whole-genome sequencing, and HiFi reads were generated for each of the four KI mouse reporter strains. An average read length of ~15 kb was obtained for each KI ranging from ~28.3 Gbp to ~35.5 Gbp HiFi reads with the coverage between 10x to 13x. The coverage was chosen as a trade-off between our desire to examine all four knock-in strains and the cost of sequencing.

### Read alignment and variant calling

The HiFi reads were aligned to the reference genome, mm10 (GRCm38.68 version) using the pbmm2(v1.4.0) (https://github.com/PacificBiosciences/pbmm2) alignment tool with default options for index and align. We used Deepvariant caller^13^ (v1.4.0) (default options with–vsc_min_fraction_indels 0.12) to identify the SNPs/INDELs. To phase the genomic variants, (haplotype assembly) WhatsHap (v1.1)^14^ with default phase options was used. We separated the SNPs and INDELs using (GATK (v4.4.0.0))^15^ because the SNPs and INDELs files are separately available in the mouse genome project. From these SNPs and INDELs, we filtered the variants (with QUAL less than 30 (QUAL30)) (-filter “QUAL < 30.0”–filter-name “QUAL30”). We compared them to the controls (described below), and removed the background SNPs/INDELs with RTG tool’s (v3.12.1)^16^ vcfeval (rtg vcfeval -f QUAL) to discover SNPs/INDELs uniquely found in the KI mouse reporter strains.

Background controls were obtained from two sources to remove naturally occurring variants found in wildtype animals. First, we used the SNPs/INDELs generated by the Mouse Genome Project from control C57BL/6NJ mouse samples with respect to the genome version GRCm38.68^17^. Since the KI mice were generated from a cross between C57BL/6Ncr and B6D2F1/J mice, we considered the genome sequences of B6D2F1/J which is a cross between C57BL/6J (B6) female and DBA/2J (D2) male, heterozygous for B6 and D2 alleles. Therefore, we took both the parental lines (C57BL/6J and DBA/2J) as controls for our analysis. The second set of controls was generated from six published PacBio datasets for C57BL/6NJ and DBA/2J. Among these, five (three C57BL/6NJ, two DBA/2J) are from Ferraj *et al*.^18^ and one is from Arslan *et al*.^19^ (C57BL/6NJ). With two (C57BL/6NJ from Ferraj *et al*.^18^) out of the six PacBio datasets, we were able to produce output from Deepvariant caller^13^ (v1.4.0), and the remaining four failed to produce Deepvariant output due to computational limitations. We therefore applied the steps from alignment through variant filtering (with QUAL30) and generated separate control SNPs and INDELs using three C57BL/6NJ datasets and one DBA/2J dataset.

### Structural variant analysis

The Mouse Genome Project did not have any data to use as controls for structural variant analysis. So, we took the six PacBio control datasets to find wildtype structural variants. Structural variant (SV) discovery was performed using the pbsv (https://github.com/PacificBiosciences/pbsv) (v2.6.2) tool (pbsv discover–tandem-repeats and pbsv call with default options). Tandem repeat annotations were obtained using the findTandemRepeats function from pbsv (https://github.com/PacificBiosciences/pbsv/tree/master/annotations/findTandemRepeats) to increase the sensitivity and recall for structural variants. The resulting structural variant VCF files were compared between control vs KIs using Truvari^20^ (truvari bench–passonly -r 1000 -p 0.01) to obtain SVs uniquely found in KI reporter strains.

### Variant effects analysis

A VCF file containing our final SNPs/INDELs or SV callset was used as input for the Ensembl Variant Effect Predictor (v102) tool^21^ (vep –everything with default option) to intersect SNP/INDELs or SV coordinates with mm10 (GRCm38) features. Based upon the variants and a type of genomic region, VEP annotates the predicted effects of the SNP/INDELs or SVs.

### Off-target analysis

Cas-OFFfinder was used to identify potential off-targets of the gRNA. The criteria of up to 5 mismatches and 1 DNA/RNA bulge were used. The total number of off-targets identified from the seven gRNAs ranged from ~3700 to ~5600. The intersections between the off-targets and the variants found in our analysis from the knock-in strains were obtained.

## Data Records

The sequencing fastq data files are available at the NCBI BioSample database under the BioProject PRJNA1075111^22–25^: NCBI Sequence Read Archive https://identifiers.org/ncbi/insdc.sra: SRP489185 (2024).

All the variant VCF data files are available at the European Variation Archive database https://identifiers.org/ena.embl:PRJEB80026 (2024)^26^.

## Technical Validation

Homozygosity of the KI reporter was confirmed by genotyping the animals of each mouse line. The DNA samples were subject to quality check prior to sequencing. The PacBio sequencing HiFi reads passed the quality control according to the PacBio analysis pipeline.

## Supplementary information

Table S1

Table S2

Table S3

Table S4

Figure S1

## Data Availability

No custom code was generated in this study.
