# Supplementary material for: Whole genome sequencing of CRISPR/Cas9-engineered NF-κB reporter mice for validation and variant discovery
Source: Sci Data. 2024 Nov 13;11:1225. doi: 10.1038/s41597-024-04064-8 (PMC11561245; doi:10.1038/s41597-024-04064-8)
Supplement: Supplementary file 5 — Figure S1 [file 41597_2024_4064_MOESM5_ESM.pdf]

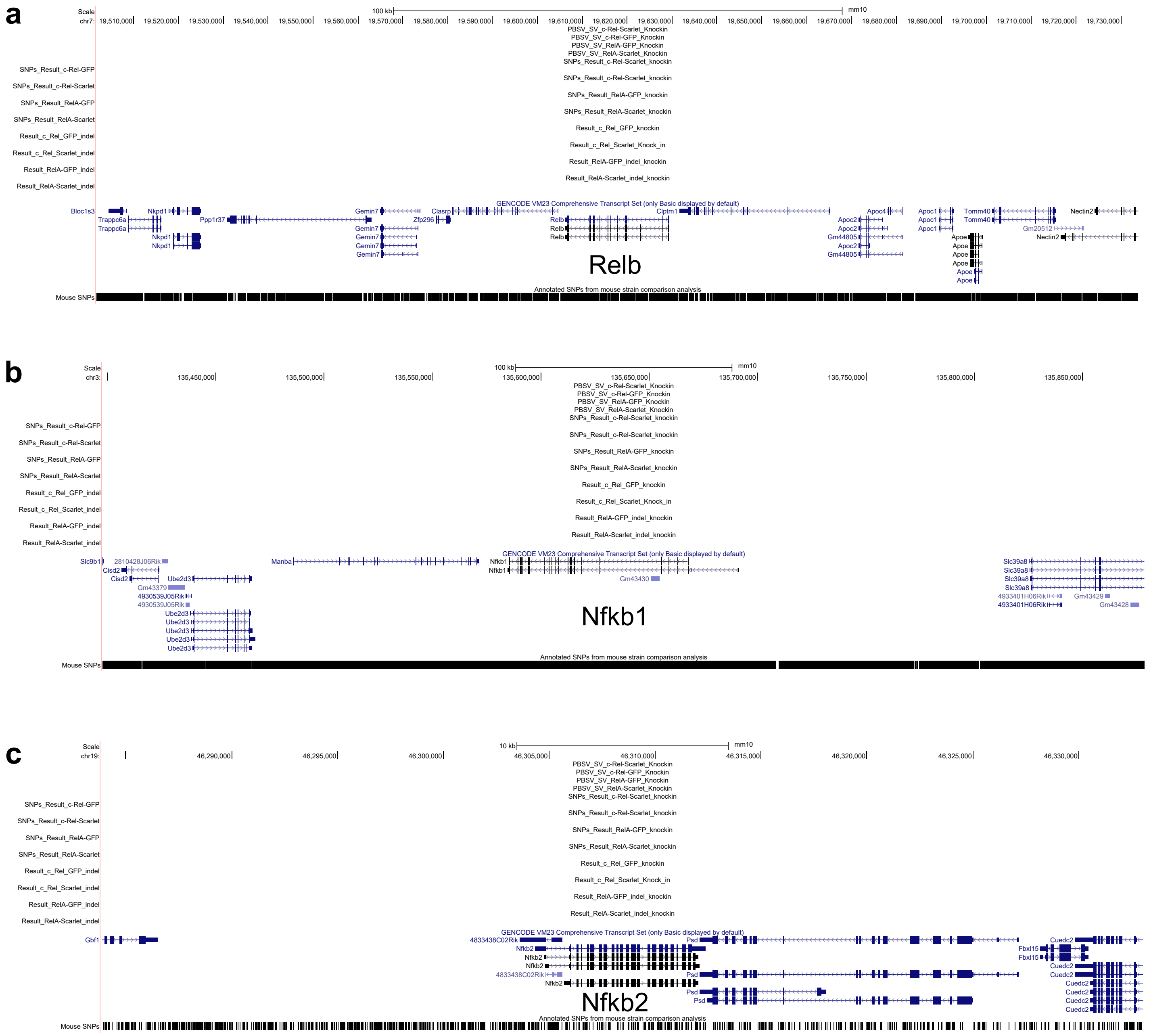

Figure S1. Whole-genome sequencing confirms that untargeted loci, Relb, NFkb1, and Nfkb2, are undisturbed in all four reporter strains.

The genome browsershots of the other loci encoding NF-kB proteins: Relb (a), Nfkb1 (b), Nfkb2 (c).
